# Wnt5a is associated with right ventricular dysfunction and adverse outcome in dilated cardiomyopathy

**DOI:** 10.1038/s41598-017-03625-9

**Published:** 2017-06-14

**Authors:** Aurelija Abraityte, Ida G. Lunde, Erik T. Askevold, Annika E. Michelsen, Geir Christensen, Pål Aukrust, Arne Yndestad, Arnt Fiane, Arne Andreassen, Svend Aakhus, Christen P. Dahl, Lars Gullestad, Kaspar Broch, Thor Ueland

**Affiliations:** 1 Research Institute of Internal Medicine, Oslo University Hospital, Rikshospitalet, Oslo Norway; 2 Faculty of Medicine, University of Oslo, Oslo, Norway; 3 Center for Heart Failure Research, University of Oslo, Oslo, Norway; 4 Institute for Experimental Medical Research, Oslo University Hospital and University of Oslo, Oslo, Norway; 5 Section of Clinical Immunology and Infectious Diseases, Oslo University Hospital, Rikshospitalet, Oslo Norway; 6 K. G. Jebsen Inflammation Research Center, University of Oslo, Oslo, Norway; 7 K. G. Jebsen Thrombosis Research and Expertise Center, University of Tromsø, Tromsø, Norway; 8 Department of Cardiothoracic Surgery, Oslo University Hospital, Rikshospitalet, Oslo Norway; 9 Department of Cardiology, Oslo University Hospital, Rikshospitalet, Oslo Norway; 10 Department of Circulation and Imaging, Faculty of Medicine, NTNU, Norwegian University of Science and Technology, Trondheim, Norway

## Abstract

The Wingless (Wnt) pathway has been implicated in the pathogenesis of dilated cardiomyopathy (DCM). To explore the role of Wnt modulators Wnt5a and sFRP3 in DCM patients we analyzed the expression of Wnt5a and sFRP3 in plasma and myocardium of DCM patients and evaluated their effects on NFAT luciferase activity in neonatal mouse cardiomyocytes. Elevated circulating Wnt5a (n = 102) was associated with increased pulmonary artery pressures, decreased right ventricular function and adverse outcome, with a stronger association in more severely affected patients. A higher Wnt5a/sFRP3 ratio (n = 25) was found in the right ventricle *vs*. the left ventricle and was correlated with NFAT activation as well as pulmonary artery pressures. Wnt5a induced NFAT activation and sFRP3 release in cardiomyocytes *in vitro*, while sFRP3 antagonized Wnt5a. Wnt5a is associated with right ventricular dysfunction and adverse outcome in DCM patients and may promote the progression of DCM through NFAT signaling.

## Introduction

Heart failure (HF) is a progressive disorder associated with high morbidity and mortality^1^. One of the most common heart conditions leading to the development of HF is dilated cardiomyopathy (DCM), characterized by cardiac chamber dilatation, reduced systolic function and is often the reason for heart transplantation (HTx)^2^. Although the management of patients with HF has improved, current treatments such as β-adrenoreceptor antagonists and inhibitors of the renin–angiotensin–aldosterone system are insufficient, at least in subgroups of the patients. Therefore, less studied pathogenic pathways that are inadequately modified by current treatments could represent new targets for therapy.

Several signaling pathways are dysregulated in the failing myocardium. These include the wingless (Wnt) signaling pathway, quiescent in the normal adult heart, but reactivated during heart disease^3^. Wnt proteins initiate signaling by binding to a receptor complex composed of a Frizzled receptor and a low-density lipoprotein receptor-related protein 5/6, resulting in different intracellular responses, involving β-catenin (canonical pathway), Ca^2+^ or other second messengers (non-canonical pathways)^3, 4^. Le Dour found evidence for decreased canonical Wnt signaling in a murine model for cardiomyopathy caused by a lamin A/C mutation^5^. Also, DKK3, a Wnt antagonist belonging to the Dickkopf family, prevented familial dilated cardiomyopathy development by activating the canonical and inhibiting the non-canonical pathway^6^. Clinical support for decreased canonical signaling is found in the study by Schumann *et al*., who demonstrated decreased β-catenin levels in patients with DCM together with enhanced levels of the Wnt antagonists secreted frizzled-related protein (sFRP) −3 and sFRP4^7^. In contrast, a recent study found that β-catenin is stabilized and translocated into the nucleus of cardiomyocytes in failing hearts from patients with both ischemic heart disease and idiopathic DCM, as well as in a murine HF model^8^. Thus, the exact role of Wnt signaling in DCM is controversial although there is an agreement that dysregulation of this pathway may be involved in HF progression.

One of the proposed non-canonical signaling pathways, the Wnt-Ca^2+^ pathway, activates calcineurin and its downstream transcription factor nuclear factor of activated T-cells (NFAT). NFAT activation is central to cardiac remodeling in HF, promoting cardiac hypertrophy, fibrosis and progression of HF in experimental models^9, 10^. Moreover, there is increased activity of the calcineurin/NFAT pathway in patients with DCM^11^. In preliminary analysis we found that the prototypical canonical Wnt ligands Wnt3a and Wnt8 were undetectable in myocardial tissue, while the canonical ligands Wnt 5a and Wnt11 were expressed at similar levels. Wnt5a has recently been shown to induce cardiomyocyte hypertrophy through c-Jun N-terminal kinase (JNK) activation^12^. Furthermore, Wnt5a activates NFAT in chondrocytes^13^ and cancer cells^14^. The role of Wnt5a in HF, including if Wnt5a interferes with NFAT signaling within the failing myocardium, is largely unknown.

We have recently demonstrated increased myocardial and circulating Wnt5a and sFRP3 in another HF population^15, 16^. Wnt5a and sFRP3 interactions have been described in multiple tissue and cells. In mice, they show a reciprocal expression pattern and antagonism during regulation of the non-canonical PCP pathway^17^. Furthermore, we found that Wnt5a induced sFRP3 release in cardiac fibroblasts at a posttranscriptional level in mouse cardiac fibroblasts^15^. sFRP3 may also antagonize Wnt5a in dendritic cells^18^, mesenchymal stromal cells^19^, melanoma cells^20^, during chondrogenesis^21^ and in the CNS^22^. To further elucidate the role of Wnt5a in HF we analyzed the expression of Wnt5a and sFRP3 in plasma and myocardium of patients with DCM. We also hypothesized that the Wnt5a/sFRP3 ratio could reflect the balance between agonist and antagonist in this system and mirror Wnt5a activity.

Our specific aims were to (i) evaluate associations between circulating levels of Wnt5a, sFRP3, their ratio and hemodynamic measures of ventricular function and adverse outcome; (ii) investigate myocardial expression of Wnt5a and sFRP3 and their ratio in the left ventricle (LV) and the right ventricle (RV) and correlations with Regulator of calcineurin (RCAN)1.4, a marker of NFAT signaling, as well as hemodynamic myocardial measures of ventricular function; (iii) examine if these Wnt modulators could interfere with NFAT signaling in cardiomyocytes.

## Results

### Wnt5a and sFRP3 are associated with cardiac function

Patient characteristics are shown in Table 1. Firstly, we determined plasma levels of Wnt5a and Wnt antagonist sFRP3 in 102 patients with idiopathic DCM. As sFRP3 is able to bind Wnt5a and inhibit its action^17–22^, we calculated a Wnt5a/sFRP3 ratio as a surrogate for Wnt5a activity. We evaluated associations between cardiac function and baseline levels of Wnt5a, sFRP3 and their ratio (Table 2). Plasma Wnt5a correlated positively with pulmonary arterial pressure, pulmonary capillary wedge pressure, left atrial area, the E/A ratio, RV end-systolic and end-diastolic volume; and negatively correlated with RV ejection fraction (RVEF). No associations were found between Wnt5a and indices of LV function or cardiac output. sFRP3 and the WNt5a/sFRP3 ratio were more modestly associated with RV function (Table 2).

**Table 1:** Characteristics of patients with idiopathic DCM (n = 102) and end-stage DCM who underwent heart transplantation (n = 25).

Variable	Plasma cohort (n = 102)	Tissue cohort (n = 25)
Age years	51 ± 14	45 ± 16
Male gender – no (%)	76 (73)	20 (80)
Body mass index (kg/m^2^)	28 ± 5	25 ± 4
Systolic BP (mm Hg)	117 ± 21	
Diastolic BP (mm Hg)	71 ± 12	
Heart rate (beats/min)	75 ± 16	79 ± 15
NYHA class I/II/III/IV	16/62/20/6	0/0/18/6
Smokers	25 (24)	12 (48)
Hypertension	18 (17)	2 (8)
Diabetes mellitus	4 (4)	2 (8)
Atrial fibrillation	18 (17)	4 (16)
Duration HF (months)	7 (3, 15)	6 (2, 10)
Hypercholesterolemia	4 (4)	
Hemoglobin (g/dl)	14.4 ± 1.5	13.8 ± 1.6
Creatinine (mmol/l)	86 ± 21	112 ± 45
NT-proBNP (pmol/l)	153 (64, 335)	426 (199, 641)
Leukocytes	7.9 ± 2.0	8.3 ± 3.0
Platelets	269 (228, 316)	229 (210, 300)
hsTnT (ng/l)	13 (10, 20)	N.A.
Cholesterol (mmol/l)	4.8 ± 1.2	3.5 (0.9)
hsCRP (mg/l)	3 (1, 8)	5 (3, 11)
Loop diuretics	74 (71)	22 (88)
Aldosterone antagonist	23 (22)	11 (44)
ACE inhibitor or ARB	101 (97)	24 (96)
β-blocker	97 (93)	22 (88)
Statins	24 (23)	3 (12)

Patient characteristics are presented as mean ± standard deviation or median (25^th^/75^th^ percentile) for continuous variables and % of cases for categorical variables. BP, blood pressure; HF, heart failure; hsCRP, high-sensitivity C-reactive protein; hsTnT, high-sensitive troponin T; NT-proBNP, N-terminal pro-brain natriuretic peptide; NYHA, New York Heart Association.

**Table 2:** Associations between plasma Wnt5a, sFRP3, the Wnt5a/sFRP3 ratio and ventricular function in patients with DCM.

Variable	All patients	Wnt5a	sFRP3	Wnt ratio
Pulmonary artery systolic pressure (mm Hg)	37 ± 12	0.35***	0.05	0.33**
Mean pulmonary artery pressure (mm Hg)	24 ± 10	0.26**	0.12	0.24*
Pulmonary capillary wedge pressure (mm Hg)	14 (8, 33)	0.25*	0.21*	0.17
Left atrial area (cm^2^)	28 ± 6	0.24*	0.31**	0.06
E/A ratio	1.31 (0.77, 2.07)	0.40***	0.34**	0.23*
Cardiac output (l/min)	4.9 ± 1.4	−0.05	−0.15	0.01
Left ventricular end-systolic volume (ml)	191 (141, 269)	0.16	0.13	0.04
Left ventricular end-diastolic volume (ml)	264 (214, 327)	0.15	0.14	0.07
Left ventricular ejection fraction (%)	27 ± 10	−0.17	−0.08	−0.14
Right ventricular end-systolic volume (ml)	110 (82, 161)	0.32**	0.23*	0.14
Right ventricular end-diastolic volume (ml)	188 (155, 241)	0.30**	0.25*	0.19
Right ventricular ejection fraction (%)	39 ± 13	−0.27*	−0.18	−0.22*

Data are given as mean ± standard deviation or median (25^th^/75^th^ percentile) depending on distribution. *p < 0.05, **p < 0.01, ***p < 0.001.

### Wnt5a and sFRP3 associations with long-term outcome

**Figure 1:**
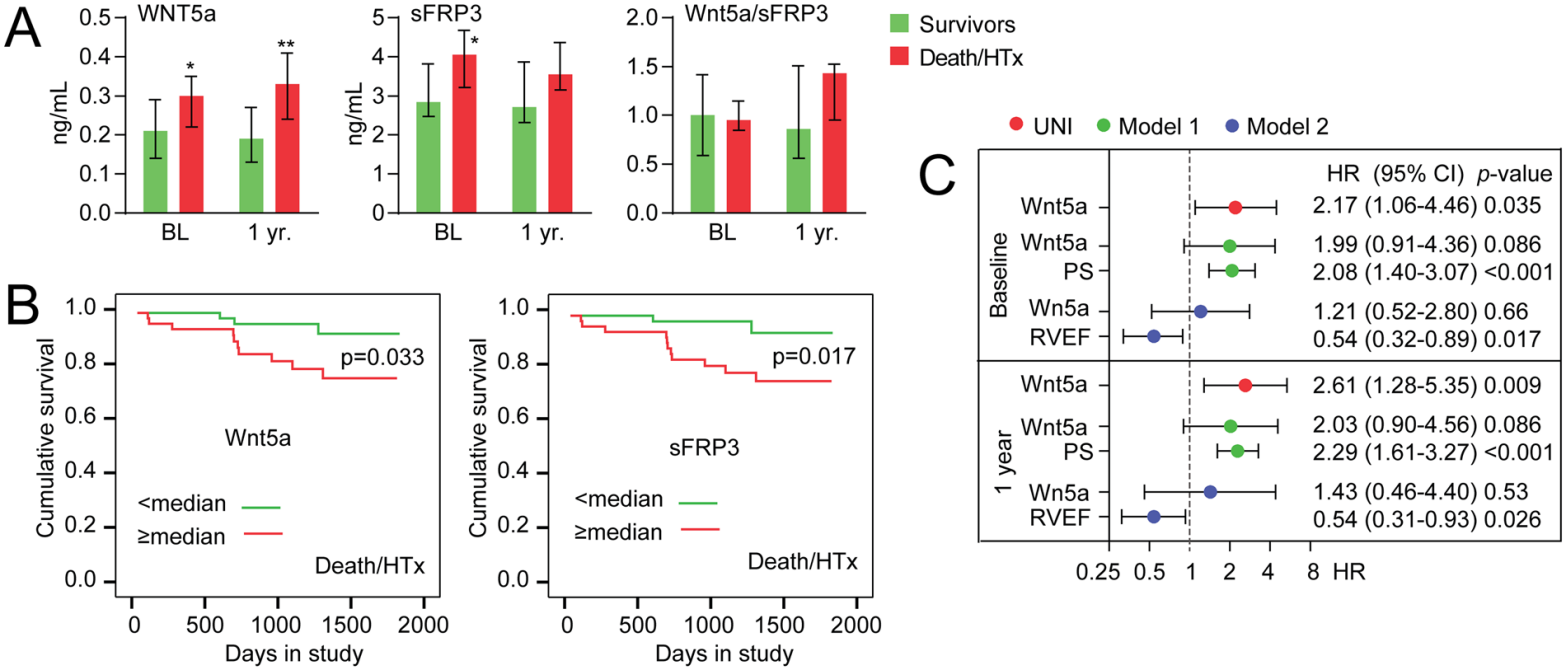
Wnt5a and sFRP3 associations with adverse outcome in patients with DCM. (**A**) Circulating Wnt5a, sFRP3 and their ratio (Wnt5a/sFRP3) in DCM patients with adverse outcome (death or HTx) *vs* survivors at baseline (BL) and 1 year follow-up. (**B**) Kaplan-Meier curves showing the cumulative incidence of mortality or HTx (n = 13) during the whole study period (median follow-up 3.3 years, 0.2–5.1), according to dichotomized levels of Wnt5a and sFRP3 at enrolment. (**C**) The relationship between Wnt5a and outcome shown as hazard ratios (HRs) and 95% confidence interval per standard deviation (SD) change with p-values for univariate (UNI), propensity score (PS) adjusted (age, gender, creatinine, NYHA and NT-proBNP) (model 1), and adjusted for RVEF (model 2) Cox hazards models. *p < 0.05, **p < 0.01 *vs*. survivors. DCM, dilated cardiomyopathy, HTx, heart transplantation; NT-proBNP, N-terminal pro-brain natriuretic peptide; NYHA, New York Heart Association; RVEF, right ventricular ejection fraction; sFRP3, secreted frizzled-related protein 3.

During a median 3.3 years follow-up, 13 patients had adverse outcome (died or had HTx). At baseline, circulating Wnt5a was 45% higher (p = 0.025) in patients who had adverse outcome compared to survivors. When the patients were reexamined after one year (n = 10, 12 to 16 months) Wnt5a was 71% higher in patients with outcome (p = 0.002) (Fig. 1A). A similar pattern was seen for sFRP3 with an increase of 43% (p = 0.01) at baseline and 31% after one year in those with adverse outcome (Fig. 1A). A modest correlation was observed between Wnt5a and sFRP3 (r = 0.23, p = 0.02). The Wnt5a/sFRP3 ratio was similar in patients with- and without outcome. Figure 1B shows Kaplan-Meier curves for associations between Wnt5a, sFRP3 and long-term mortality. Cox regression analysis revealed a significantly increased risk of adverse outcome of 2.2 at baseline and 2.6 after one year follow-up, per standard deviation increase in Wnt5a (Fig. 1C). Further adjustment using a propensity score (age, gender, creatinine, NYHA and NT-proBNP) had limited influence on Wnt5a’s hazard rate both at baseline and after one year (Fig. 1C). However, replacing the propensity score with RVEF dissipated the association between Wnt5a and adverse outcome.

### Interactions between Wnt5a and sFRP3 levels and indices of ventricular function

**Figure 2:**
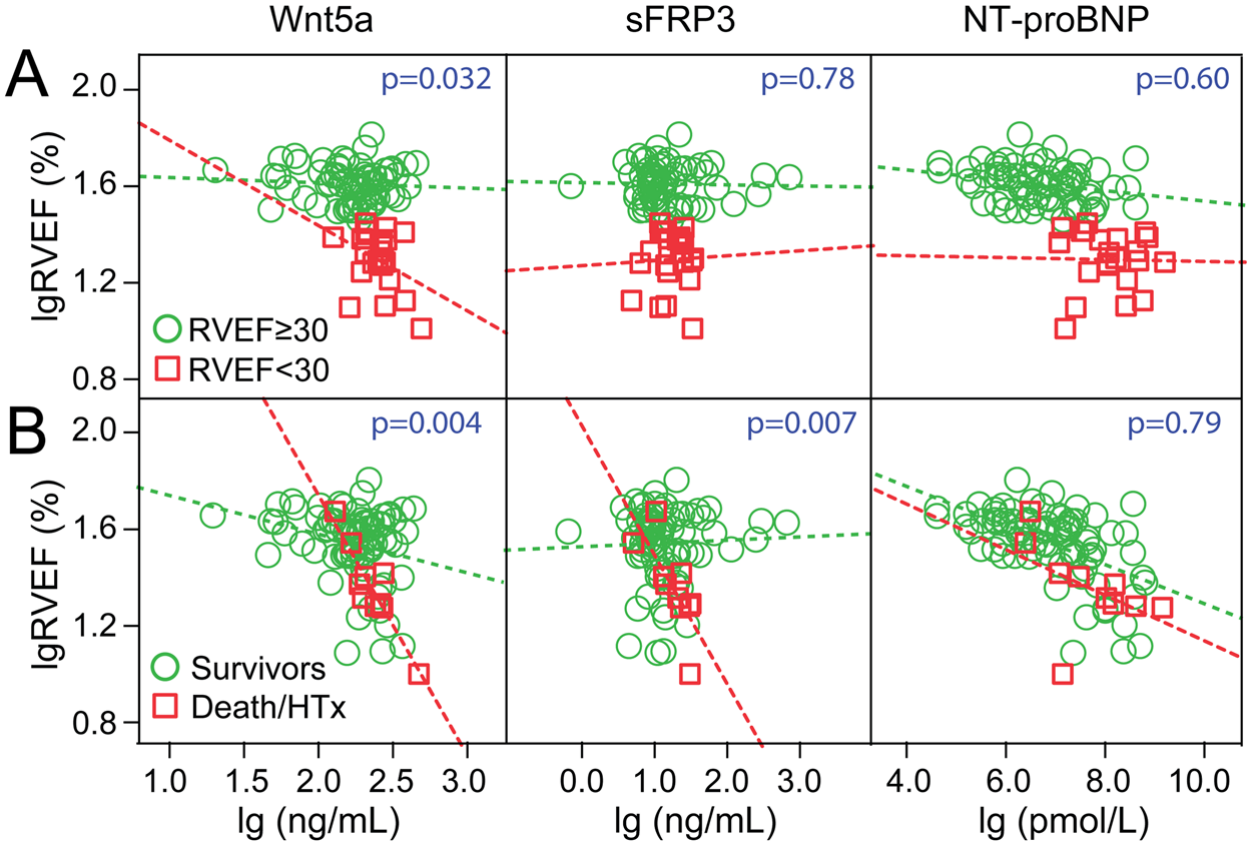
Interactions between Wnt5a and sFRP3 levels and indices of ventricular function. Interaction analysis between Wnt5a, sFRP3, NT-proBNP and RVEF (**A**) in patients with impaired RV function (i.e. RVEF < 30%, n = 22) and (**B**) in patients with adverse outcome (n = 13). Interaction analysis was performed including covariate (e.g. Wnt5a), fixed factor (e.g. RVEF with cut-off) and their interaction term. The p-value of the interaction term is shown in blue. HTx, heart transplantation; NT-proBNP, N-terminal pro-brain natriuretic peptide; RVEF, right ventricular ejection fraction; sFRP3, secreted frizzled-related protein 3.

RV failure may constitute a “common final pathway” in the progression of HF^23^. Therefore, we evaluated whether the association between the level of Wnt5a and declining ventricular function was more prominent in patients with poor prognosis or more severe RV dysfunction. Patients with severe RV dysfunction (i.e. RVEF < 30%^24^) had markedly elevated Wnt5a at baseline (RVEF < 30 median 25^th^/75^th^ percentile 0.29 [0.21, 0.31] *vs*. RVEF ≥ 30 0.18 [0.13, 0.25]; p = 0.001). As shown in Fig. 2A, the association between Wnt5a and RVEF was only present in patients with severe RV dysfunction. This pattern was not present for sFRP3. Furthermore, significant interactions between Wnt5a, sFRP3 and RVEF were present when comparing the regression lines in survivors *vs*. Death/HTx (Fig. 2B). This indicates that an increase in Wnt5a in patients with adverse outcome was associated with a larger decline in RV function. In contrast, although NT-proBNP was strongly associated with RVEF, an adverse outcome status or the degree of RV dysfunction did not interact with this association.

### Wnt5a and sFRP3 expression in the myocardium of DCM patients

**Figure 3:**
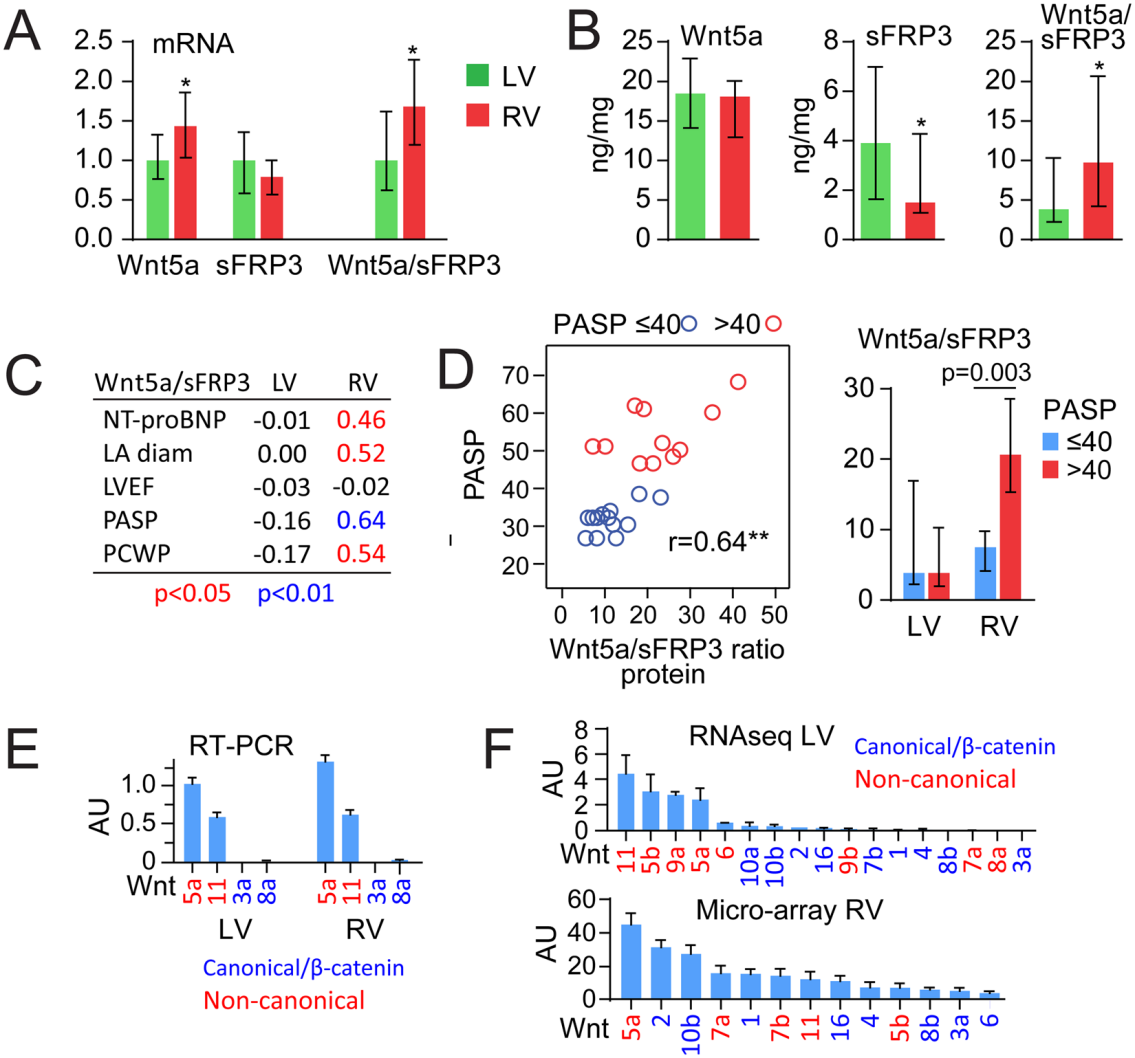
Wnt5a and sFRP3 expression in the myocardium of DCM patients. (**A**) mRNA and (**B**) protein levels of Wnt5a, sFRP3 and their ratio (Wnt5a/sFRP3) in the LV and RV myocardial tissue of DCM patients (n = 25). (**C**) Correlations between Wnt5a/sFRP3 ratio and NT-proBNP (n = 25), LA diameter (n = 20), LVEF (n = 23), PASP (n = 24) and PCWP (n = 24) in the LV and RV of DCM patients. (**D**) Correlation between Wnt5a/sFRP3 ratio and PASP in the RV and comparison of the Wnt5a/sFRP3 ratio within the LV and RV between DCM patients with pulmonary hypertension (i.e. PASP > 40 mm Hg, n = 11) and without it (i.e. PASP ≤ 40 mm Hg, n = 13). (**E**) mRNA expression of typical canonical and non-canonical Wnt ligands in the LV and RV of DCM patients obtained in preliminary studies. (**F**) mRNA expression profiles of other Wnt ligands in the RV of DCM patients (GSE29819) as well as some RNAseq data from the LV (GSE71613) obtained from the GEO repository. DCM, dilated cardiomyopathy; LA, left atrial; LV, left ventricle, LVEF, left ventricular ejection fraction; NT-proBNP, N-terminal pro-brain natriuretic peptide; PASP, pulmonary artery systolic pressure; PCWP, pulmonary capillary wedge pressure; RV, right ventricle; sFRP3, secreted frizzled-related protein 3.

To further elucidate associations between Wnt5a, sFRP3, their ratio and myocardial function, we determined their mRNA and protein expression in LV and RV myocardial tissue of 25 patients with DCM. As shown in Fig. 3A, the mRNA expression of Wnt5a and the Wnt5a/sFRP3 ratio was higher in the RV than in the LV, while sFRP3 expression was similar in both ventricles. Furthermore, sFRP3 protein expression was lower and consequently, the Wnt5a/sFRP3 ratio was higher in the RV (Fig. 3B). A modest correlation between RV tissue Wnt5a and sFRP3 protein expression was observed (r = 0.35, p = 0.089) with no association in LV (r = 0.11, p = 0.56). The RV, but not the LV, Wnt5a/sFRP3 protein ratio correlated positively with NT-proBNP levels, left atrial diameter, pulmonary capillary wedge pressure and in particular pulmonary artery systolic pressure (PASP) (Fig. 3C). Patients with PASP > 40 mm Hg (median 51), indicating pulmonary hypertension, had a markedly higher Wnt5a/sFRP3 ratio (Fig. 3D). In preliminary studies we evaluated mRNA levels of typical canonical (Wnt3a, Wnt8a) and non-canonical (Wnt5a and Wnt11) Wnt ligands in myocardial tissue from DCM patients and found that the non-canonical ligands were expressed with the highest levels of Wnt5a, while the canonical Wnt3a and Wnt8 were hardly expressed (CT values from RT-PCR > 35) (Fig. 3E). A similar pattern with expression of non-canonical Wnt ligands was observed when evaluating expression patterns from GEO repositories although some differences were observed in microarrays from the RV of DCM patients and RNAseq from the LV (Fig. 3F).

### Wnt5a and sFRP3 effects on NFAT signaling

**Figure 4:**
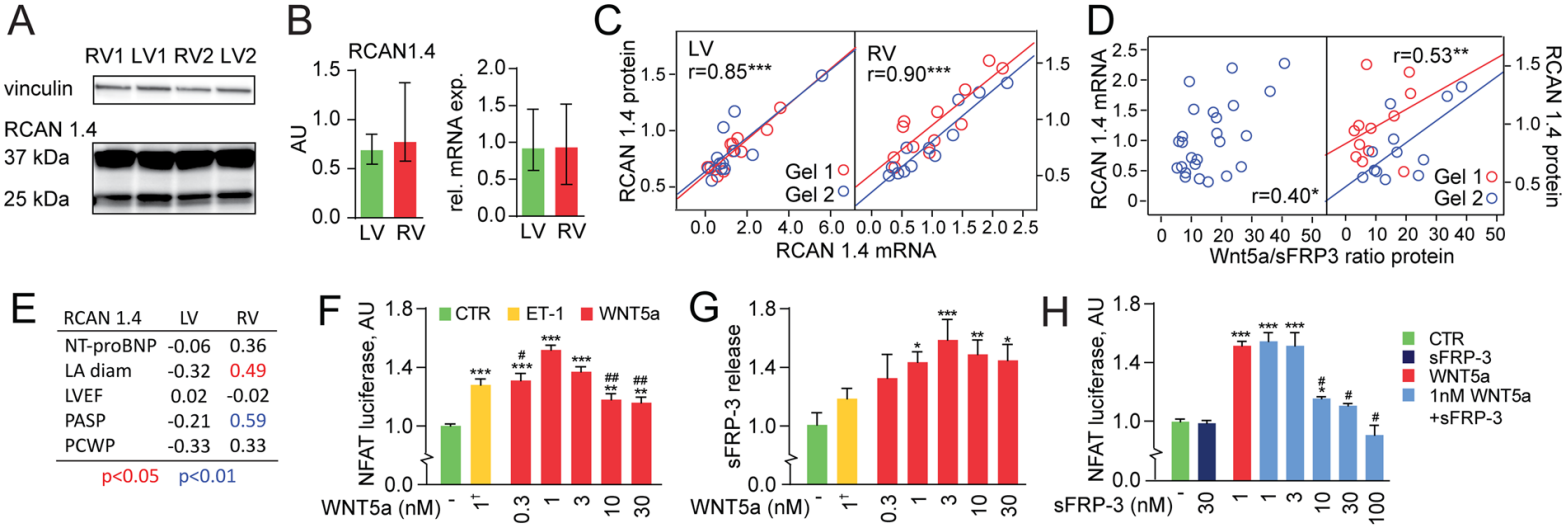
Wnt5a and sFRP3 effects on NFAT signaling. (**A**) Representative immunoblots of RCAN1.4 and loading control Vinculin in the LV and RV of two DCM patients. (**B**) mRNA and protein levels of RCAN1.4 in the LV and RV myocardial tissue of DCM patients (n = 25). (**C**) Correlation between RCAN1.4 mRNA and protein levels (adjusting for gel with partial correlation). (**D**) Correlation between the Wnt5a/sFRP3 ratio and RCAN1.4 mRNA and protein levels (adjusting for gel with partial correlation). (**E**) Correlations between RCAN1.4 and NT-proBNP (n = 25), LA diameter (n = 20), LVEF (n = 23), PASP (n = 24) and PCWP (n = 24) in the LV and RV of DCM patients. (**F**) NFAT luciferase activity in neonatal mouse cardiomyocytes after treatment with endothelin-1 (ET-1) and increasing concentrations of recombinant Wnt5a. (**G**) A relative sFRP3 release in culture medium from F. (**H**) NFAT luciferase activity after treatment with recombinant sFRP3, Wnt5a or both. *p < 0.05, **p < 0.01, ***p < 0.001 vs. control (CTR), ^#^p < 0.05, ^##^p < 0.01 *vs* 1 nM Wnt5a, ^†^1 µM endothelin-1 (ET-1). DCM, dilated cardiomyopathy; LA, left atrial; LV, left ventricle, LVEF, left ventricular ejection fraction; NFAT, nuclear factor of activated T-cells; NT-proBNP, N-terminal pro-brain natriuretic peptide; PASP, pulmonary artery systolic pressure; PCWP, pulmonary capillary wedge pressure; RCAN1.4, regulator of calcineurin 1 isoform 4; RV, right ventricle; sFRP3, secreted frizzled-related protein 3.

Since NFAT has been shown to promote cardiac hypertrophy and the progression of DCM^9^, we evaluated mRNA and protein expression of RCAN1.4, a target of NFAT signaling. The human RCAN1 gene comprises seven exons, with expression of exons 4–7 giving rise to the RCAN1.4 protein. Expression of exon 4 is extremely sensitive to calcineurin-NFAT activity due to a cluster of 15 NFAT binding sites in its promoter^25^. Figure 4A shows representative immunoblots of RCAN1.4 in the LV and RV of DCM patients. There were no differences in RCAN1.4 mRNA or protein levels between the LV and RV (Fig. 4B), but mRNA and protein levels correlated strongly in both ventricles (Fig. 4C). Furthermore, there was a positive correlation between Wnt5a/sFRP3 protein ratio and both mRNA and protein levels of RCAN1.4 in the RV (Fig. 4D). Moreover, RCAN1.4 mRNA levels in the RV, but not in the LV were positively correlated with left atrial diameter and PASP (Fig. 4E).

We next investigated if Wnt5a and sFRP3 affected NFAT signaling *in vitro*. As shown in Fig. 4F, recombinant mouse Wnt5a activated NFAT in cardiomyocytes from NFAT-luciferase reporter mice in a more pronounced manner than did high-dose endothelin-1, which was used as positive control. Moreover, Wnt5a stimulation enhanced sFRP3 release from the cardiomyocytes (Fig. 4G), suggesting that a negative feedback loop, regulating Wnt signaling, could be operating in these cells. Indeed, sFRP3 stimulation suppressed Wnt5a-induced NFAT activity (Fig. 4H), while sFRP3 alone did not have an effect on NFAT activation level.

## Discussion

Wnt signaling has recently been implicated in maladaptive cardiac remodeling and cardiomyopathy through activation of both canonical and non-canonical pathways^8, 26–28^. Herein we extend and lend clinical support to previous findings, showing that Wnt5a was associated with RV dysfunction and adverse outcome in patients with DCM. In particular, (i) elevated circulating Wnt5a was associated with increased pulmonary artery pressures, decreased RV function and adverse outcome, with a stronger association in more severely affected patients. (ii) A higher Wnt5a/sFRP3 ratio was found in the RV *vs*. the LV and was correlated with NFAT activation and pulmonary artery pressures. Finally, (iii) Wnt5a induced NFAT activation and sFRP3 release in mouse cardiomyocytes, while sFRP3 antagonized Wnt5a. Our findings suggest that Wnt5a signaling could activate NFAT in RV in advanced DCM, with sFRP3 as a potential counteracting regulator.

In systolic HF, increased LV filling pressures may cause pulmonary hypertension^23, 29^. This pressure overload leads to RV hypertrophy and subsequently progressive contractile dysfunction as well as decompensated RV failure characterized by increased filling pressures and diastolic dysfunction^23, 29^. Indeed, both RV function and pulmonary hypertension are strong predictors of adverse outcome in DCM, independent of LVEF^30^. Our finding that elevated circulating Wnt5a was associated with several indices of RV function suggests that there is a link between Wnt5a and the deterioration of RV function in advanced DCM. Furthermore, Wnt5a was associated with adverse outcome in survival analysis, but this association was mitigated when RVEF was included in the model, further supporting that the relationship between Wnt5a and HF severity may reflect its association with declining RV function. These finding support and extend our recent study in a different HF population of mixed etiology where serum Wnt5a was elevated in HF patients and associated with hemodynamic, neurohormonal, and clinical measures of disease severity^15^.

Inhibition of Wnt5a signaling with a homologous peptide fragment reduces infarct expansion and prevents the development of HF in animal models^31, 32^. Natural regulators of Wnt signaling include multiple secreted modulators, such as sFRP3, which can bind Wnt5a and antagonize its action in different cells^17–22^, as also demonstrated for cardiomyocytes in the present study. We have previously observed markedly enhanced circulating and myocardial mRNA expression of sFRP3 in clinical and experimental HF^16^. Our finding in the present study, that sFRP3 is released upon Wnt5a stimulation *in vitro* and to some degree mirrors the associations between Wnt5a, RV function and outcome *in vivo*, may suggest that enhanced sFRP3 in HF could reflect active Wnt signaling and represent a feedback loop to limit Wnt5a activity. In mouse cardiac fibroblasts, we have also observed that Wnt5a may induce sFRP3 release in cardiac fibroblasts at a posttranscriptional level^15^. If a similar mechanism is present in cardiomyocytes, this could perhaps partly explain the inconsistent mRNA and protein expression of Wnt5a and sFRP3 in myocardial tissue. Importantly, however, the Wnt5a/sFRP3 ratio was increased for both mRNA and protein. Caution is needed when interpreting the biological significance of ratios in clinical and experimental medicine. However, when analyzing the balance between agonist and antagonist in relevant pathways, agonist and antagonist ration could reflect bioactivity as has been shown for the IL6 and TNF activity^33, 34^, and our findings herein showing sFRPs inhibits Wnt5a induced NFAT activity suggest that this may also be the case for the Wnt5a pathway.

Alternatively, sFRP3 could also have Wnt independent effects and /or influence Wnt signaling by other mechanisms than antagonizing Wnt ligands, including interactions with other Wnt antagonists which could promote Wnt signaling, binding to frizzled receptors directly and inhibiting Wnt signaling and finally, facilitating Wnt signaling by presentation of Wnt to its receptor^35^. However, with regards to NFAT activation, the addition of sFRP3 alone had no effect on NFAT activity, but addition in combination with the maximum NFAT activity stimulating dose of Wnt5a, dose dependently antagonized this effect, favoring an antagonistic effect of sFRP3. This supports the importance of a balance between Wnt5a and sFRP3 and that like the TNF/TNFR ratio may determine TNF activity, Wnt5a/sFRP3 ratio may influence Wnt5a activity. However, further studies are needed to elucidate the molecular mechanisms in the interaction between Wnt5a and sFRP3.

Furthermore, while the association between Wnt5a and RVEF was particularly strong in patients with more severe RV dysfunction, the lack of this association for sFRP3 could indicate that it is unable to adequately antagonize Wnt5a in DCM patients with end-stage disease. Notwithstanding, in preliminary studies we found a similar expression level of Wnt11 and Wnt5a and some interactions between Wnt11 and sFRP3 on diffusion have been demonstrated^36^. The expression profiles from the GEO repository support mainly expression of non-canonical Wnt ligands in human myocardial tissue, although many of these ligands can affect both canonical and non-canonical pathways. In addition, the canonical ligands Wnt2 and Wnt10b were abundantly expressed in microarray data from the RV, but not RNAseq from the LV of DCM patients. However, apart from Wnt 1^37^, Wnt3a^38^ and 8^37, 39^, which were expressed at low levels, we found little information on interactions between other Wnt ligands than Wnt5a and sFRP3. Nonetheless, we cannot exclude that sFRP3 could interact with other Wnt ligands or have Wnt independent effects on other cellular processes relevant in the progression of HF.

The Wnt5a/sFRP3 ratio correlated with increased right arterial pressures with a particularly high ratio in patients with pulmonary hypertension as also observed in HF patients of mixed etiology^15^. Wnt5a has been extensively examined in pulmonary hypertension and in diseases characterized by pulmonary fibrosis, often complicated by pulmonary hypertension and RV dysfunction^40^. The exact role in pulmonary hypertension is controversial and Wnt5a has been shown to both reduce^41, 42^ and enhance^43^ hypoxia stimulated growth and β-catenin activation. Furthermore, both beneficial^44^ and adverse^43^ effects on RV function have been shown in mouse pulmonary hypertension models, the latter is more in line with the Wnt5a as a hypertrophic signal, required for the activation of protein synthesis and cardiomyocyte hypertrophy^12, 45^. In COPD and idiopathic pulmonary fibrosis, pulmonary Wnt5a was almost exclusively secreted by fibroblasts, had pro-fibrotic effects and affected the balance between canonical and non-canonical Wnt signaling^46–48^. The effects of Wnt5a on fibrosis are highly relevant as ECM remodeling and fibrosis is important features in pathological remodeling and hypertrophy. Furthermore, we have recently demonstrated that Wnt5a may induce IL-6 and TIMP-1 in an extracellular signal regulated kinase 1/2 (ERK1/2) dependent manner, which might promote myocardial inflammation and fibrosis, and thereby contribute to HF progression. Our findings support experimental studies demonstrating that specific pathways are selectively involved in RV remodeling with reactivation of the fetal gene program^49^, including the Wnt pathway^50^. Thus, dysregulated Wnt signaling in the RV, secondary to pulmonary hypertension, may underlie development of RV dysfunction, and this may partly reflect an imbalance between Wnt5a and its antagonist sFRP3.

Enhanced NFAT signaling directly promotes cardiac hypertrophy, ventricular dilatation and the development of experimental HF^10^ and DCM^11^. Wnt5a has been shown to induce NFAT signaling in chondrocytes^13^ and cancer cells^14^. The correlation between the Wnt5a/sFRP3 ratio and the NFAT target gene RCAN1.4 in RV myocardium from patients with end-stage DCM as well as NFAT activation by Wnt5a in cardiomyocytes from NFAT-luciferase reporter mice, suggests that Wnt5a-induced NFAT signaling could be active and involved in the pathogenesis of DCM.

Limitations to our study include a relatively small patient cohort with a low number of adverse events. Moreover, associations between myocardial and circulating levels of Wnt modulators and clinical parameters do not necessarily translate to causal relationship. Finally, our study was restricted to patients with DCM and further studies are needed to clarify if our findings apply to the general HF population.

## Conclusion

Our study found that patients with DCM are characterized by dysregulated systemic and myocardial Wnt signaling involving Wnt5a and the Wnt antagonist sFRP3. High circulating Wnt5a correlated with increased pulmonary artery pressures, decreased RV function and adverse outcome. A higher Wnt5a/sFRP3 ratio in the RV correlated with NFAT activation and pulmonary artery pressures. Finally, Wnt5a induced NFAT activation and sFRP3 release in mouse cardiomyocytes, while sFRP3 antagonized Wnt5a. Our findings suggest that dysregulated Wnt5a signaling could promote the progression of DCM through NFAT signaling.

## Methods

### Patients and study procedures

*Study population 1* (Table 1) consisted of 102 patients with a diagnosis of idiopathic DCM and has been described in detail previously^51^. At baseline, the patients underwent physical examination; blood tests; echocardiography; cardiac magnetic resonance imaging (MRI) and right-sided cardiac catheterization. One year after inclusion, the patients were invited for reassessment. The patients were subsequently followed-up through the Norwegian National Population Register and our heart transplant database for mortality and HTx, respectively. The primary outcome was the composite of all-cause mortality and HTx.

*Study population 2* (Table 1) consisted of 25 patients with DCM and end-stage HF. Human myocardial LV and RV tissue samples were obtained from still-beating hearts of patients undergoing HTx, were snap-frozen in liquid nitrogen for mRNA and protein analyses and stored at −80 °C until use.

The human protocols conformed to the Declaration of Helsinki and were approved by the Norwegian Regional Committee for Research Ethics (REK). Informed written consent was obtained from all patients. Mouse protocols were reviewed and approved by the Norwegian National Animal Research Committee and conformed to the NIH Guide for the Care and Use of Laboratory Animals.

### Echocardiography, MRI and right-sided heart catheterization

Echocardiography was performed with Vivid 7 or E9 ultrasound scanners (GE Vingmed Ultrasound, Horten, Norway), using phased array transducers. 2D parameters and conventional Doppler measurements were obtained according to current recommendations^52, 53^. MRI was performed with Siemens 1.5 Tesla scanners (Siemens Avanto and Siemens Sonata, Siemens Medical Systems, Erlangen, Germany). Seven millimeter thick short axis slices covering the entire left and right ventricles were acquired and endocardial borders were traced manually at a PACS work station (Sectra Medical Systems AB, Linköping, Sweden). Ventricular volumes and ejection fractions were calculated by short axis slice summation. Right-sided heart catheterization was performed using a Swan–Ganz pulmonary artery thermodilution catheter (Baxter Health Care Corp, Santa Ana, CA). Intracardiac pressures were recorded, and cardiac output was measured by the thermodilution technique.

### Blood sampling and biochemical analyses

Peripheral blood samples were obtained in the non-fasting state at inclusion and at one year follow-up. Blood was collected in vacutainers containing ethylenediamine tetraacetic acid and immediately centrifuged at 2,000 g for 20 minutes. Platelet-poor plasma was aliquoted, stored at −80 °C and thawed < 3 times before analysis. N-terminal pro-B-type natriuretic peptide (NT-proBNP) was determined by an electrochemiluminescence immunoassay (Roche proBNP II, Roche Diagnostics, Basel, Switzerland). Levels of high sensitivity C-reactive protein (hsCRP) were determined on a MODULAR Analytical platform, P800 module (Roche Diagnostics) using a particle-enhanced immunoturbidimetric assay (Tina-Quant CRP Gen.3, Roche Diagnostics). Plasma and myocardial extract levels of Wnt5a and sFRP3 were measured by enzyme immunoassays (EIAs) from Cusabio (Wuhan, China) and R&D Systems (Stillwater, MN, USA), respectively. All samples were analyzed in duplicate. The intra- and inter-assay coefficient of variation for the EIAs was < 10%.

### NFAT-luciferase reporter assay

NFAT-luciferase mice _ENREF_9^9^, kindly provided by Dr. Jeffery D. Molkentin (Cincinnati Children’s Hospital Medical Center, Cincinnati, OH), carry nine copies of NFAT-binding sites from the interleukin-4 promoter, upstream of the luciferase gene. Primary ventricular cardiomyocyte cultures from neonatal (one-three days old) NFAT-luciferase reporter mice were prepared as described^54^. Cell cultures from three separate isolations were used for experiments. Cardiomyocytes were maintained in serum-free medium 24 hours prior to treatment with recombinant Wnt5a, sFRP3 (R&D Systems, Minneapolis, MN, USA) or endothelin-1 (used as positive control, Sigma-Aldrich, St. Louis, MO, USA) for 24 hours, washed twice with PBS and harvested for luciferase activity quantification according to the luciferase assay protocol (Promega, Madison, WI, USA). Luminescence from duplicates was quantified on a Victor 3 1420 Multilabel Counter (PerkinElmer, MA). Cell culture medium was collected for cell viability analyses (ToxiLight, Lonza Group Ltd, Basel, Switzerland) and measurements of sFRP3 release measured by EIAs (R&D Systems; Stillwater, MN, USA).

### RNA extraction and real-time PCR

Total RNA was extracted from human myocardial tissue using TRIzol (Invitrogen, San Diego, CA, USA) and RNeasy®Micro kit (Qiagen, Hilden, Germany) according to the manufacturer’s instructions. RNA concentration was measured with a spectrophotometer (NanoDrop ND-1000, Thermo Fisher Scientific, Waltham, MA, USA). cDNA was synthesized using High Capacity cDNA Reverse Transcription Kit (Thermo Fisher Scientific). Quantification of gene expression was performed using Power SYBR Green Master Mix (Applied Biosystems/Life Technologies Corporation, Carlsbad, CA, USA) and mRNA sequence-specific PCR primers on ABI Prism 7500 Real-Time PCR Systems (Applied Biosystems). Primer sequences are available upon request. Glyceraldehyde 3-phosphate dehydrogenase (GAPDH) was used as reference gene.

### Protein analysis

Frozen myocardial tissue was supplied with ice-cold PBS-based lysis buffer containing 1% Triton X-100 (Sigma-Aldrich, St. Louis, MO, USA), 0.1% Tween-20 (Sigma-Aldrich), protease and phosphatase inhibitors (Complete EDTA-free and PhosStop tablets, Roche Diagnostics, Germany, respectively). Samples were homogenized twice for 30 sec with a Polytron 1200, left on ice for 30 min and centrifuged at 20 000 g for 10 min at 4 °C. Supernatants were stored at −70 °C. SDS-PAGE and immunoblotting were performed according to the Criterion BIO-RAD (CA, USA) protocol using 10 μg protein. Blots were blocked in 5% dry-milk (BIO-RAD) over night and incubated with antibodies diluted in 5% dry-milk for one hour. Primary antibodies were anti-RCAN1 (D6694) and anti-vinculin (V9131) from Sigma-Aldrich, with horse radish peroxidase (HRP)-conjugated secondary antibodies (#1031 and #4030) from Southern Biotechnology. Blots were developed using the ECL Plus Western Blotting Detection System (GE Healthcare, UK) and Las-4000 mini (Fujifilm, Japan). Data processing was performed using Image J (NIH, MD, USA).

### Statistics

Values are presented as mean ± standard deviation or median (25^th^, 75^th^ percentile) depending on distribution as evaluated by the Kolmogorov Smirnov test. Differences between groups were compared with Students t-test or the Mann Whitney U test depending on distribution and χ^2^ test for categorical variables. In the paired situation, Wilcoxon matched pairs test was used. The association between dichotomized levels of Wnt5a, sFRP3 and all-cause mortality/HTx was evaluated by Kaplan–Meier analysis with log-rank test while continuous variables are expressed as log transformed Z-score for Cox-regression. Associations between variables were assessed by Spearman correlation or with regression analysis on log transformed values depending on distributions. All statistical analyses were performed with the Statistical Package for Social Sciences version 22 software (SPSS Inc. Chicago, IL). Two-sided probability values were considered significant at p < 0.05.

## References

[1] Liu, L. & Eisen, H. J. Epidemiology of heart failure and scope of the problem. *Cardiology clinics* **32**, 1–8 vii (2014). 10.1016/j.ccl.2013.09.009 24286574

[2] Sanbe A (2013). Dilated cardiomyopathy: a disease of the myocardium. Biological & pharmaceutical bulletin.

[3] Dawson K, Aflaki M, Nattel S (2013). Role of the Wnt-Frizzled system in cardiac pathophysiology: a rapidly developing, poorly understood area with enormous potential. The Journal of physiology.

[4] ter Horst P, Smits JF, Blankesteijn WM (2012). The Wnt/Frizzled pathway as a therapeutic target for cardiac hypertrophy: where do we stand?. Acta physiologica (Oxford, England).

[5] Le Dour, C. *et al*. Decreased WNT/beta-catenin signalling contributes to the pathogenesis of dilated cardiomyopathy caused by mutations in the lamin a/C gene. *Human molecular genetics* (2017). 10.1093/hmg/ddw389 PMC6075603 28069793

[6] Hou, N. *et al*. Transcription Factor 7-like 2 Mediates Canonical Wnt/beta-Catenin Signaling and c-Myc Upregulation in Heart Failure. *Circulation. Heart failure* **9** (2016). 10.1161/CIRCHEARTFAILURE.116.003010 PMC5060009 27301468

[7] Schumann H, Holtz J, Zerkowski HR, Hatzfeld M (2000). Expression of secreted frizzled related proteins 3 and 4 in human ventricular myocardium correlates with apoptosis related gene expression. Cardiovascular research.

[8] Lu, D. *et al*. Dkk3 prevents familial dilated cardiomyopathy development through Wnt pathway. *Laboratory investigation; a journal of technical methods and pathology* (2015). 10.1038/labinvest.2015.145 26641069

[9] Wilkins BJ (2004). Calcineurin/NFAT coupling participates in pathological, but not physiological, cardiac hypertrophy. Circ Res.

[10] Tang M (2010). Proteasome functional insufficiency activates the calcineurin-NFAT pathway in cardiomyocytes and promotes maladaptive remodelling of stressed mouse hearts. Cardiovascular research.

[11] Diedrichs H, Chi M, Boelck B, Mehlhorn U, Schwinger RH (2004). Increased regulatory activity of the calcineurin/NFAT pathway in human heart failure. European journal of heart failure.

[12] Hagenmueller M (2014). Dapper-1 is essential for Wnt5a induced cardiomyocyte hypertrophy by regulating the Wnt/PCP pathway. FEBS letters.

[13] Bradley EW, Drissi MH (2010). WNT5A regulates chondrocyte differentiation through differential use of the CaN/NFAT and IKK/NF-kappaB pathways. Molecular endocrinology (Baltimore, Md.).

[14] Griesmann H (2013). WNT5A-NFAT signaling mediates resistance to apoptosis in pancreatic cancer. Neoplasia (New York, N.Y.).

[15] Abraityte, A. *et al*. Wnt5a is elevated in heart failure and affects cardiac fibroblast function. *Journal of molecular medicine* (2017). 10.1007/s00109-017-1529-1 28357477

[16] Askevold ET (2014). The cardiokine secreted Frizzled-related protein 3, a modulator of Wnt signalling, in clinical and experimental heart failure. Journal of internal medicine.

[17] Qian D (2007). Wnt5a functions in planar cell polarity regulation in mice. Developmental biology.

[18] Holtzhausen A (2015). Melanoma-Derived Wnt5a Promotes Local Dendritic-Cell Expression of IDO and Immunotolerance: Opportunities for Pharmacologic Enhancement of Immunotherapy. Cancer immunology research.

[19] Yamada A, Iwata T, Yamato M, Okano T, Izumi Y (2013). Diverse functions of secreted frizzled-related proteins in the osteoblastogenesis of human multipotent mesenchymal stromal cells. Biomaterials.

[20] Ekstrom EJ, Sherwood V, Andersson T (2011). Methylation and loss of Secreted Frizzled-Related Protein 3 enhances melanoma cell migration and invasion. PloS one.

[21] Liu W (2008). Coordinated molecular control of otic capsule differentiation: functional role of Wnt5a signaling and opposition by sfrp3 activity. Growth factors (Chur, Switzerland).

[22] Li B (2013). Wingless-type mammary tumor virus integration site family, member 5A (Wnt5a) regulates human immunodeficiency virus type 1 (HIV-1) envelope glycoprotein 120 (gp120)-induced expression of pro-inflammatory cytokines via the Ca2^+^/calmodulin-dependent protein kinase II (CaMKII) and c-Jun N-terminal kinase (JNK) signaling pathways. The Journal of biological chemistry.

[23] Voelkel NF (2006). Right ventricular function and failure: report of a National Heart, Lung, and Blood Institute working group on cellular and molecular mechanisms of right heart failure. Circulation.

[24] Pavlicek M (2011). Right ventricular systolic function assessment: rank of echocardiographic methods vs. cardiac magnetic resonance imaging. European journal of echocardiography: the journal of the Working Group on Echocardiography of the European Society of Cardiology.

[25] Oh M, Dey A, Gerard RD, Hill JA, Rothermel BA (2010). The CCAAT/enhancer binding protein beta (C/EBPbeta) cooperates with NFAT to control expression of the calcineurin regulatory protein RCAN1–4. The Journal of biological chemistry.

[26] Okada K (2015). Wnt/beta-Catenin Signaling Contributes to Skeletal Myopathy in Heart Failure via Direct Interaction With Forkhead Box O. Circulation. Heart failure.

[27] Xi XH (2015). Activation of Wnt/beta-catenin/GSK3beta signaling during the development of diabetic cardiomyopathy. Cardiovascular pathology: the official journal of the Society for Cardiovascular Pathology.

[28] Zhang M (2015). Calcium/calmodulin-dependent protein kinase II couples Wnt signaling with histone deacetylase 4 and mediates dishevelled-induced cardiomyopathy. Hypertension.

[29] Moraes DL, Colucci WS, Givertz MM (2000). Secondary pulmonary hypertension in chronic heart failure: the role of the endothelium in pathophysiology and management. Circulation.

[30] Hirashiki A (2014). Prognostic value of pulmonary hypertension in ambulatory patients with non-ischemic dilated cardiomyopathy. Circulation journal: official journal of the Japanese Circulation Society.

[31] Laeremans H (2011). Blocking of frizzled signaling with a homologous peptide fragment of wnt3a/wnt5a reduces infarct expansion and prevents the development of heart failure after myocardial infarction. Circulation.

[32] Hermans, K. *et al*. UM206, a Peptide Fragment of Wnt5a, Attenuates Adverse Remodeling after Myocardial Infarction in Swine. *The FASEB Journal* **29** (2015).

[33] Aukrust P (1994). Serum levels of tumor necrosis factor-alpha (TNF alpha) and soluble TNF receptors in human immunodeficiency virus type 1 infection–correlations to clinical, immunologic, and virologic parameters. The Journal of infectious diseases.

[34] Aukrust P (1999). Cytokine network in congestive heart failure secondary to ischemic or idiopathic dilated cardiomyopathy. The American journal of cardiology.

[35] Bovolenta P, Esteve P, Ruiz JM, Cisneros E, Lopez-Rios J (2008). Beyond Wnt inhibition: new functions of secreted Frizzled-related proteins in development and disease. Journal of cell science.

[36] Mii Y, Taira M (2009). Secreted Frizzled-related proteins enhance the diffusion of Wnt ligands and expand their signalling range. Development (Cambridge, England).

[37] Leyns L, Bouwmeester T, Kim SH, Piccolo S, De Robertis EM (1997). Frzb-1 is a secreted antagonist of Wnt signaling expressed in the Spemann organizer. Cell.

[38] Boland GM, Perkins G, Hall DJ, Tuan RS (2004). Wnt 3a promotes proliferation and suppresses osteogenic differentiation of adult human mesenchymal stem cells. Journal of cellular biochemistry.

[39] Wang S, Krinks M, Lin K, Luyten FP, Moos M (1997). Frzb, a secreted protein expressed in the Spemann organizer, binds and inhibits Wnt-8. Cell.

[40] Kolb TM, Hassoun PM (2012). Right ventricular dysfunction in chronic lung disease. Cardiology clinics.

[41] Takahashi J, Orcholski M, Yuan K (2016). & de Jesus Perez, V. PDGF-dependent beta-catenin activation is associated with abnormal pulmonary artery smooth muscle cell proliferation in pulmonary arterial hypertension. FEBS letters.

[42] Yu XM (2013). Wnt5a inhibits hypoxia-induced pulmonary arterial smooth muscle cell proliferation by downregulation of beta-catenin. American journal of physiology. Lung cellular and molecular physiology.

[43] Kang Z (2016). Ponatinib attenuates experimental pulmonary arterial hypertension by modulating Wnt signaling and vasohibin-2/vasohibin-1. Life sciences.

[44] Jin Y (2015). Wnt5a attenuates hypoxia-induced pulmonary arteriolar remodeling and right ventricular hypertrophy in mice. Experimental biology and medicine.

[45] He J, Cai Y, Luo LM, Wang R (2015). Expression of Wnt and NCX1 and its correlation with cardiomyocyte apoptosis in mouse with myocardial hypertrophy. Asian Pacific journal of tropical medicine.

[46] Baarsma HA (2017). Noncanonical WNT-5A signaling impairs endogenous lung repair in COPD. The Journal of experimental medicine.

[47] Rydell-Tormanen, K. *et al*. Aberrant nonfibrotic parenchyma in idiopathic pulmonary fibrosis is correlated with decreased beta-catenin inhibition and increased Wnt5a/b interaction. *Physiological reports* **4** (2016). 10.14814/phy2.12727 PMC4823602 26997628

[48] Vuga LJ (2009). WNT5A is a regulator of fibroblast proliferation and resistance to apoptosis. American journal of respiratory cell and molecular biology.

[49] Lowes BD (1997). Changes in gene expression in the intact human heart. Downregulation of alpha-myosin heavy chain in hypertrophied, failing ventricular myocardium. The Journal of clinical investigation.

[50] Urashima T (2008). Molecular and physiological characterization of RV remodeling in a murine model of pulmonary stenosis. American journal of physiology. Heart and circulatory physiology.

[51] Broch K (2015). Soluble ST2 reflects hemodynamic stress in non-ischemic heart failure. International journal of cardiology.

[52] Lang RM (2005). Recommendations for chamber quantification: a report from the American Society of Echocardiography’s Guidelines and Standards Committee and the Chamber Quantification Writing Group, developed in conjunction with the European Association of Echocardiography, a branch of the European Society of Cardiology. Journal of the American Society of Echocardiography: official publication of the American Society of Echocardiography.

[53] Galderisi M (2011). Recommendations of the European Association of Echocardiography: how to use echo-Doppler in clinical trials: different modalities for different purposes. European journal of echocardiography: the journal of the Working Group on Echocardiography of the European Society of Cardiology.

[54] Lunde IG, Kvaloy H, Austbo B, Christensen G, Carlson CR (2011). Angiotensin II and norepinephrine activate specific calcineurin-dependent NFAT transcription factor isoforms in cardiomyocytes. Journal of applied physiology (Bethesda, Md.: 1985).

